# Novel enterocin E20c purified from *Enterococcus hirae* 20c synergised with ß-lactams and ciprofloxacin against *Salmonella enterica*

**DOI:** 10.1186/s12934-020-01352-x

**Published:** 2020-05-04

**Authors:** Preeti Sharma, Muzamil Rashid, Sukhraj Kaur

**Affiliations:** Department of Microbiology, Guru Nanak Dev University, Amritsar, Punjab India

**Keywords:** Bacteriocin, Lactic acid bacteria, Antimicrobial agents, Synergistic effect, Multi-drug resistant pathogens

## Abstract

**Background:**

An increasing rate of antibiotic resistance among Gram-negative bacterial pathogens has created an urgent need to discover novel therapeutic agents to combat infectious diseases. Use of bacteriocins as therapeutic agents has immense potential due to their high potency and mode of action different from that of conventional antibiotics.

**Results:**

In this study, a novel bacteriocin E20c of molecular weight 6.5 kDa was purified and characterized from the probiotic strain of *Enterococcus hirae*. E20c had bactericidal activities against several multidrug resistant (MDR) Gram-negative bacterial pathogens. Flow cytometry and scanning electron microscopy studies showed that it killed the *Salmonella enterica* cells by forming ion-permeable channels in the cell membrane leading to enhanced cell membrane permeability. Further, checkerboard titrations showed that E20c had synergistic interaction with antibiotics such as ampicillin, penicillin, ceftriaxone, and ciprofloxacin against a ciprofloxacin- and penicillin-resistant strain of *S. enterica.*

**Conclusion:**

Thus, this study shows the broad spectrum antimicrobial activity of novel enterocin E20c against various MDR pathogens. Further, it highlights the importance of bacteriocins in lowering the minimum inhibitory concentrations of conventional antibiotics when used in combination.

## Background

The crisis of antibiotic resistance has been acknowledged as a global health emergency by WHO [1]. Concomitant to increasing rates of antibiotic resistance, the discovery of novel antimicrobials has stalled. According to a recent report, most of the new antimicrobials currently in clinical trials are the derivatives of the existing antibiotics [2]. Thus, their modes of action are similar to the parent drug that offers a short term solution to the problem of antibiotic resistance. Hence, novel therapeutic options for treating MDR infections are urgently required. Bacteriocins are considered as the next wave of protein antibiotics owing to their unique modes of action such as disruption of cell membrane integrity, inhibition of protein production, DNA replication and septum formation [3]. The applications of bacteriocins are being explored in different areas, such as healthcare, food preservation, and veterinary medicines [4]. Bacteriocins such as nisin and pediocin purified from lactic acid bacteria (LAB) have evoked commercial interest. Nisin has shown therapeutic effects for the treatment of mastitis in both human [5] and animal studies [6]. Apart from their use as standalone therapeutics, bacteriocin-antibiotic combinations have tremendous value in terms of decreasing the minimum inhibitory concentration (MIC) of antibiotics. For example, thuricin CD reduced the MIC of various antibiotics against *Clostridium* spp. [7]; and nisin showed synergistic interaction with various antibiotics against methicillin-resistant *Staphylococcus aureus* and vancomycin resistant enterococci [8]. This strategy will, in turn, reduce the probability of development of antibiotic resistance among pathogens [9].

*Salmonella*-related enteric infection is a significant health problem worldwide that poses a substantial economic burden to both developed and under-developed countries [10]. Every year, 11–20 million people become sick, and 128,000 to 161,000 people die due to typhoid fever [11]. The antibiotics commonly used for the treatment of typhoid fever belong to the classes ß-lactam and fluoroquinolone. The fast-emerging resistance to quinolones among *Salmonella* spp. has necessitated the use of other antimicrobials such as ceftriaxone, a third generation cephalosporin and azithromycin [12]. Recently sporadic cases of ceftriaxone- or azithromycin-resistant *S. typhi* have also been reported. Therefore, *Salmonella* has been classified as a high priority pathogen for the development of novel antimicrobials [13, 14]. A bacteriocin-antibiotic combination is an innovative approach to lower the dose of currently used antibiotics thereby decreasing the selective pressure that leads to the emergence of resistant bacterial strains. Thus, in this study we have purified and characterized a low molecular weight enterocin, E20c from a probiotic strain of *Enterococcus hirae* 20c that was previously isolated from healthy human vaginal swab samples [15]. Further, we studied the mechanism of antimicrobial activity of E20c and the interaction of E20c with conventionally used antibiotics against *S. enterica*.

## Results

### Purification and characterization of E20c

**Fig. 1:**
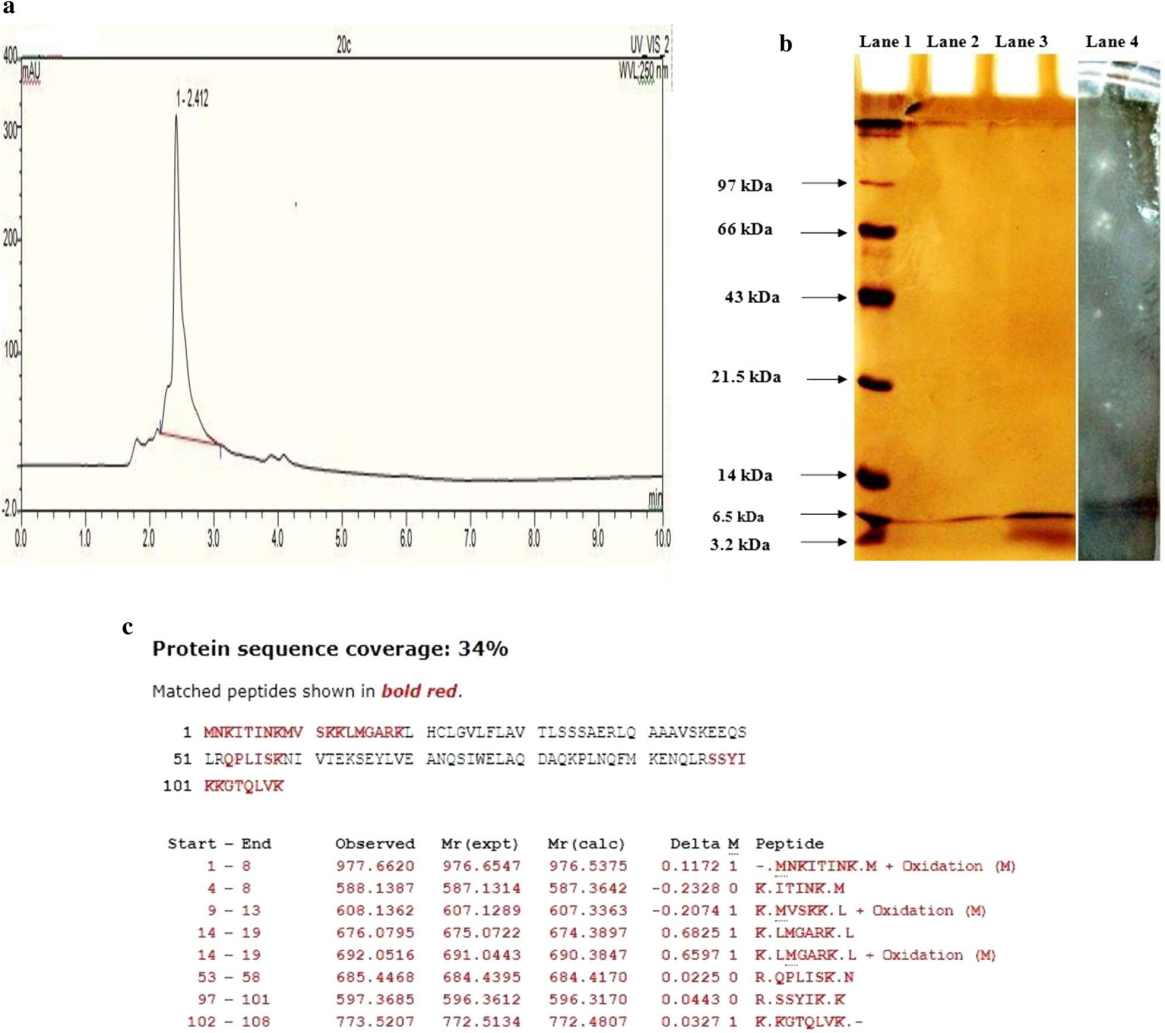
Purification and characterization of E20c. **a** U-HPLC chromatogram of the purified E20c. **b** SDS-PAGE of purified E20c. **Lane 1**: molecular weight marker. **Lane 2**: cation-exchange fraction eluted with 0.4 mM NaCl-containing sodium acetate buffer. **Lane 3**: cation-exchange fraction eluted with 0.2 mM NaCl-containing sodium acetate buffer **c** PMF analysis of E20c. The PMF followed by MASCOT search showed the matched amino acid residues (in bold red) of the peptide fragments with the hypothetical protein of *E. faecalis* following NCBI BLASTsearch

E20c was purified from the cell free culture supernatant (CS) of *E. hirae* 20c by ammonium sulphate precipitation followed by cation-exchange chromatography. Cation-exchange chromatography resulted in a 2-fold increase in the specific activity of E20c (Table 1) with the final yield percentage of 1.62. The active fractions were concentrated by lyophilization and subjected to U-HPLC and SDS PAGE analysis to check the purity of the protein. A single peak was observed in the U-HPLC chromatogram (Fig. 1a). The resolution of the concentrated active fractions on SDS-PAGE yielded a single band with a molecular weight of approximately 6.5 kDa (Fig. 1b; Lane 2). Simulataneously, a lane of agar gel was cut and subjected to agar gel overlay assay. A clear zone corresponding to 6.5 kDa band was obtained against the indicator strain *S. enterica* (Fig. 1b). Further, to characterize the protein band, it was cut, trypsin-digested and subjected to matrix-assisted laser desorption-ionization time-of-flight (MALDI TOF/TOF) mass spectrometry (MS) for protein identification. Peptide mass fingerprinting (Pmf) analysis of the fragments obtained was performed by Matrix Science Mascot UK software, and significant (p < 0.05) result was obtained. The analysis revealed eight peptides that shared similarity with 34% of the hypothetical protein of *E. faecalis* with significant (p < 0.05) coverage score of 102 (Fig. 1c).

**Table 1:** Purification of enterocin from *E. hirae* 20c

Purification step	Total volume (ml)	Activity (AU/ml)	Total activity^a^ (AU)	Total protein (mg)	Specific activity^b^ (AU/mg)	Increase in specific activity	Yield (%)
CS	1000	4266	4.2 × 10^6^	250	16800	1	100
Ammonium sulphate ppt.	50	17066	8.5 × 10^5^	21	40476.2	2.41	20.24
Cation-exchange fraction	1	68266	6.8 × 10^4^	2	34000	2.02	1.62

^a^Total activity = activity × total volume of sample used at each purification step

^b^Specific activity = total activity/total protein

### Antimicrobial activity of purified enterocins

Purified E20c had antimicrobial activities against various Gram-positive and Gram-negative pathogens viz. *S. enterica*, *Shigella flexneri*, *Escherichia coli, Streptococcus pyogenes* and *Listeria monocytogenes.* On the other hand, it did not inhibit any of the tested commensal lactobacilli spp. isolated from the stool samples of healthy children, and other tested pathogenic bacteria viz. *Vibrio cholerae*, *Mycobacterium smegmatis, S. aureus*, and *Pseudomonas aeruginosa* (Table 2).

**Table 2:** Antimicrobial activity of E20c against various indicator bacterial strains

Indicator Bacteria	Zone of inhibition (mm)
	E20c
*S. enterica* Microbial Type Culture Collection (MTCC) 733	16 ± 0.1
*Es. coli* MTCC 119	13 ± 0.12
*Sh. flexneri* MTCC 1457	15 ± 0.2
*Lis. monocytogenes* MTCC 657	14 ± 0.03
*V. cholerae* MTCC 3906	–^a^
*M. smegmatis* MTCC 6	–
*St. pyogenes* MTCC 1927	13 ± 0.11
*Staph. aureus* MTCC 96	–
*P. aeruginosa* MTCC 741	–
*L. plantarum* L14	–
*L. fermentum* L32	–
*L. pentosus* S45	–
*L. fermentum* L13	–
*L. plantarum* L12	–
*L. fermentum* L18	–
*L. casei* S49	–

Zones of inhibition (mm) of E20c against various indicator strains were determined by using agar gel diffusion assay. The results are the mean ± SD of three independent experiments

–^a^ No zone of inhibition

### Physico-chemical characterization of purified E20c

The thermostability of E20c was determined at different temperature treatments for different time periods. Results (Table 3) showed that the antimicrobial activity of E20c remained stable till 100 °C treatment for 60 min. However, at the autoclaving temperature, its activity was completely abrogated. Further, the purified E20c remained active at pH ranging from 2 to 8; maximum antimicrobial activity was observed at pH 4 and 6.

**Table 3:** Physico-chemical characterization of E20c

Physico-chemical parameter	Treatment	Zone of inhibition (mm)
	Untreated control	16 ± 0.11
Temperature	60 °C (30 min)	16 ± 0.12
	60 °C (60 min)	14 ± 0.10
	80 °C (30 min)	14 ± 0.22
	80 °C (60 min)	14 ± 0.08
	100 °C (15 min)	14 ± 0.14
	100 °C (30 min)	13 ± 0.15
	100 °C (60 min)	13 ± 0.15
	121 °C (15 min)	–*
pH	2	10 ± 0.17
	4	16 ± 0.11
	6	16 ± 0.04
	8	13 ± 0.24
	10	–
Enzymes (1 mg/ml)	Proteinase K	–
	Pepsin	–
	Trypsin	–
	Lipase	16 ± 0.12

Zone of inhibition (mm) of E20c was determined by using agar gel diffusion assay against *S. enterica*. The results are the mean ± SD of three independent experiments preformed in triplicates

–, No zone of inhibition observed

The susceptibility of E20c to various enzymes was also determined. Results showed that treatment of E20c with proteinase K, pepsin and trypsin resulted in complete abrogation of the antimicrobial activity. On the other hand, lipase treatment of E20c had no effect on the antimicrobial activity of E20c (Table 3).

### Hemolytic activity

**Fig. 2:**
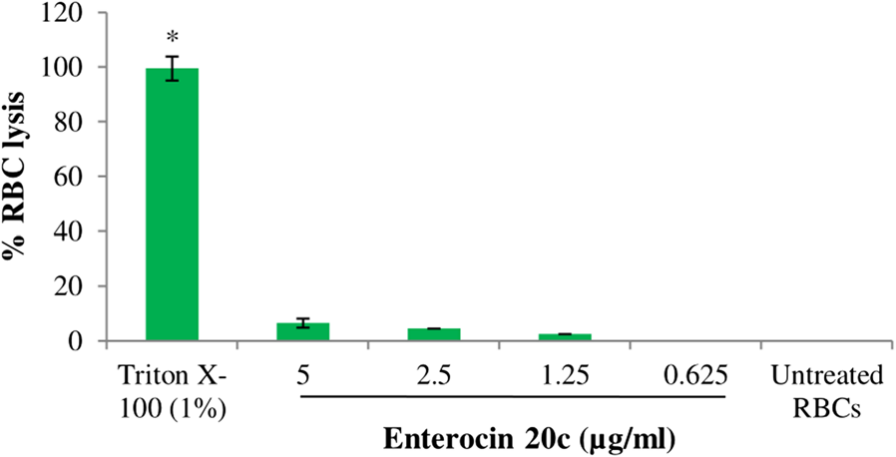
Hemolytic activity of E20c. Enterocin at different concentrations was incubated with human RBCs in PBS for 1 h. RBCs suspended in PBS were used as untreated control. Error bars represent ± SD of three independent experiments performed in triplicates. All groups were compared to the untreated control and asterisks show significant difference (p < 0.001) from untreated control

The toxicity of E20c was determined by testing its hemolytic activity against human red blood cells (RBCs). E20c at the highest concentration of 5 µg/ml did not cause any significant (p < 0.001) hemolysis of RBCs as compared to the phosphate buffer saline (PBS)-treated negative contol. On the other hand, treatment of RBCs with 1% Triton X-100 resulted in 98% hemolysis (Fig. 2).

### Minimum inhibitory concentration (MIC) and time-kill assay

**Fig. 3:**
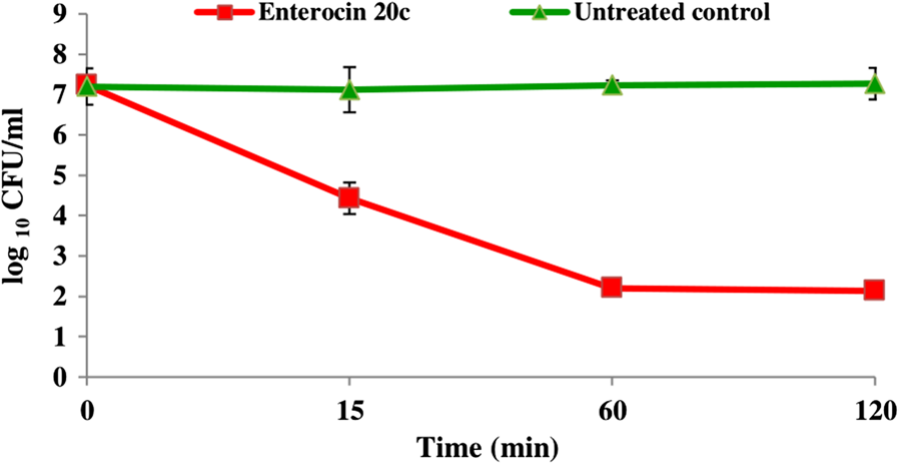
Time-kill assay of E20c against *S. enterica*. The *S. enterica* cells were treated with 2× MIC of E20c i.e. 1 µg/ml and plated on nutrient agar plates at different time points. Error bars are representative of ± SD of the three independent experiments performed in triplicates

The MIC of E20c for *S. enterica* was determined by broth microdilution assay that showed that E20c inhibited the visible growth of *S. enterica,* and the MIC was calculated as 0.5 µg/ml. Further, to determine the bactericidal mode of antimicrobial activity of E20c, time-kill assay of enterocin at 2× MIC i.e. 1 µg/ml concentration was performed against *S. enterica*. Results showed that the treatment of *S. enterica* cells with E20c resulted in 2.7 and 5.0 log10 reduction in CFU/ml at15 and 60 min, respectively (Fig. 3).

### Effect of E20c on the cell membrane permeability

**Fig. 4:**
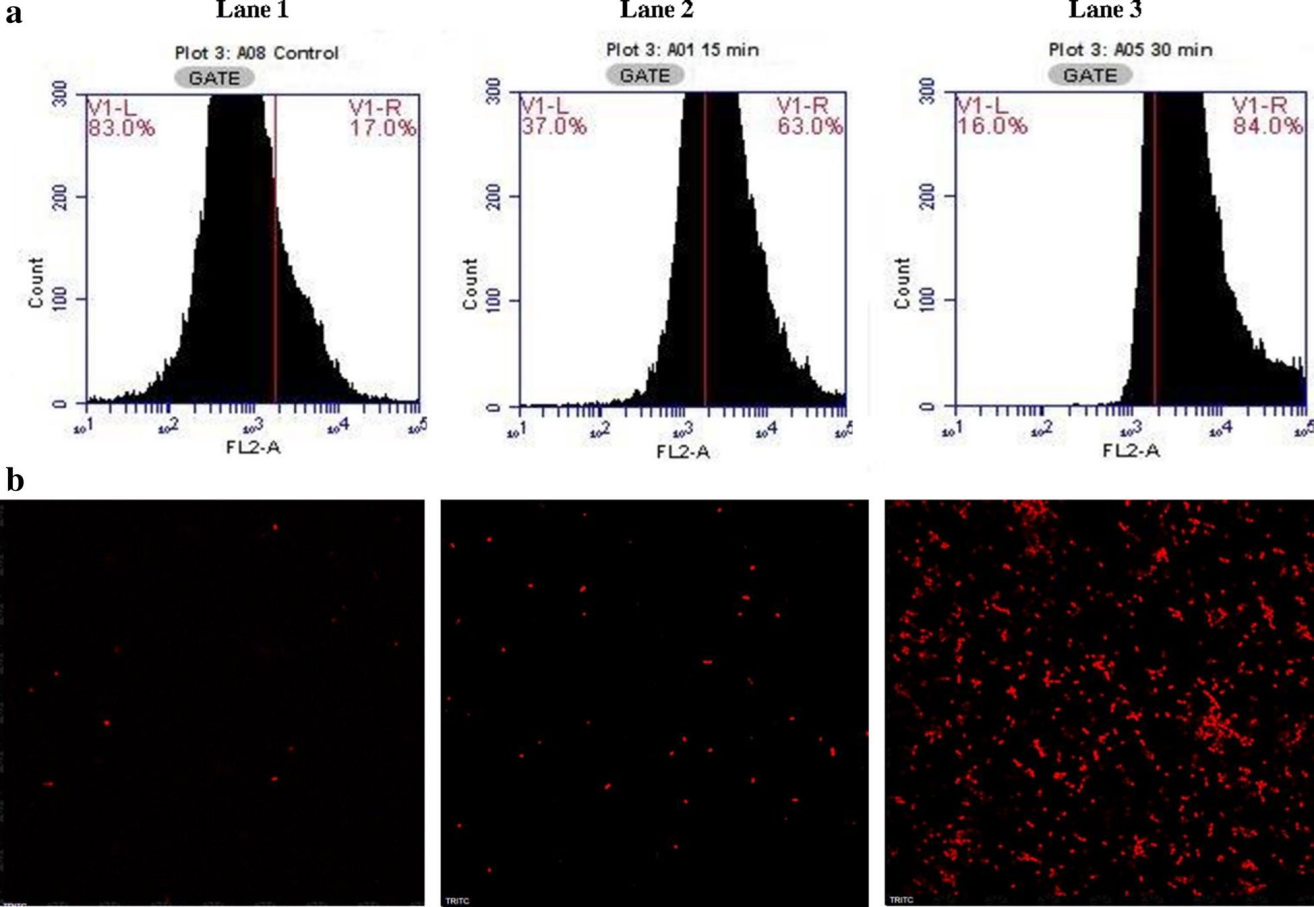
**a** Histograms showing fluorescence of PI-stained enterocin-treated/untreated *S. enterica* cells **b** confocal microscopic images depicting PI fluorescence of enterocin-treated/untreated *S. enterica* cells. **Lane 1** shows fluorescence of untreated *S. enterica* cells. **Lane 2** and **Lane 3** show fluorescence after 15 and 30 min of enterocin treatments, respectively. *S. enterica* cells in the mid-log phase were suspended in PBS and treated with 1 µg/ml of E20c for 15 and 30 min. Thereafter, the cells were stained with PI and the fluorescence was determined in flow cytometer. Data is represented as histograms with counted bacterial events displayed on y axis and increase in fluorescence on x axis

The membrane damaging effect of E20c on *S. enterica* cells was determined by flow cytometry after staining with DNA intercalating dye, propidium iodide (PI) that stains only the cells having damaged membranes. Treatment of *S. enterica* cells with E20c resulted in increase in the PI fluorescence of cells from 17% at 0 h to 63% after 15 min and to 84% at 30 min (Fig. 4a). The membrane damaging effect of E20c on *S. enterica* cells was simultaneously visualized by confocal microscopy. The images (Fig. 4b) of E20c-treated *S. enterica* cells stained with PI showed an exponential increase in the number of PI-fluorescent cells with increase in the treatment time as compared to the untreated cells.

### Scanning electron microscopy (SEM) studies

**Fig. 5:**
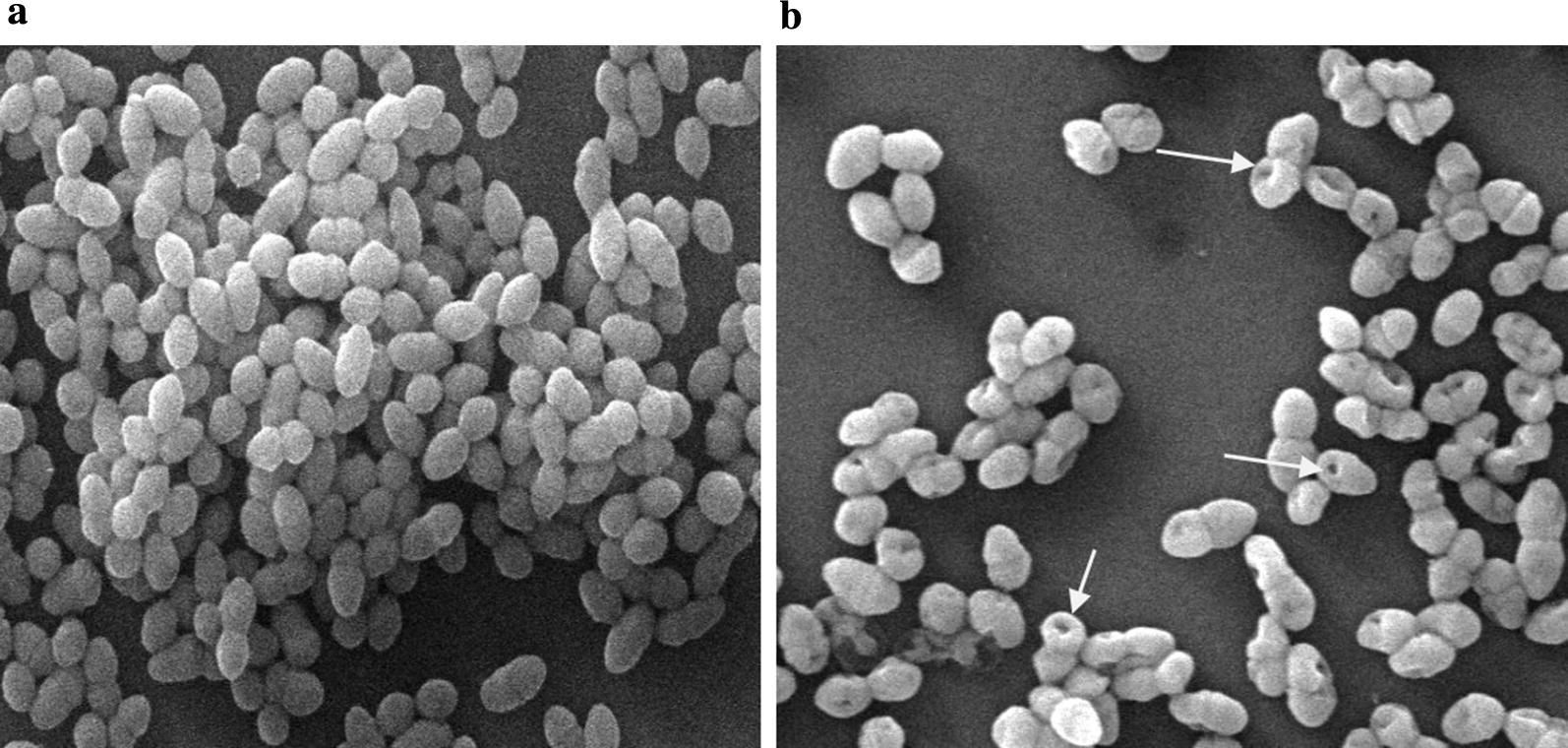
Scanning electron microscopy images of E20c-treated/untreated *S. enterica* cells at 15000X magnification **a** untreated *S. enterica* cells, **b***S. enterica* cells treated with 1 µg/ml of E20c for 60 min

Further, the effect of E20c on the cell surface of *S. enterica* cells was visualized by electron microscopy. SEM images showed that the cell surface morphology of untreated *S. enterica* cells was smooth and continuous with good structural integrity (Fig. 5a). On the other hand, E20c treatment of *S. enterica* cells resulted in the shrinkage of their volume and appearance of surface indentations probably due to the loss of cell turgidity or cell membrane damage caused by the action of E20c.

### Efflux of potassium ions

**Fig. 6:**
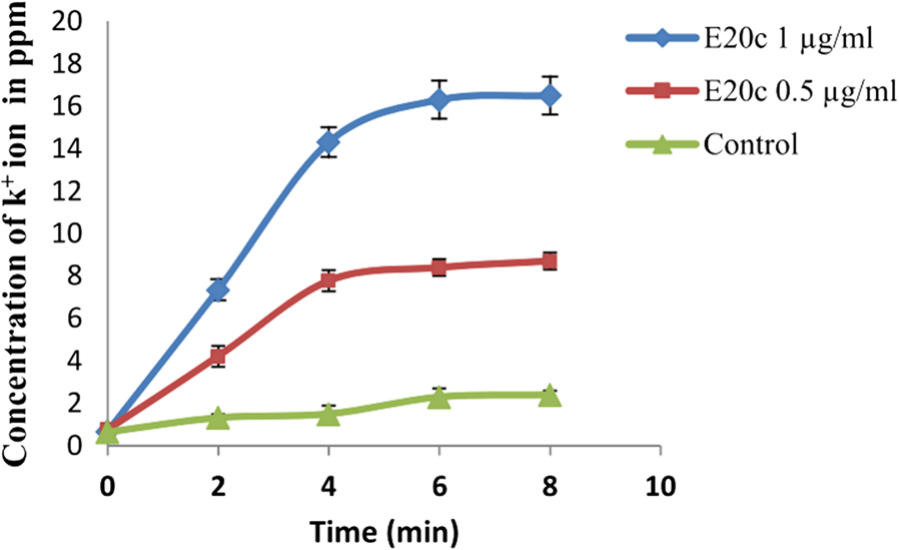
Efflux of potassium ions from *S. enterica* cells in response to the treatment of different concentrations of E20c. *S. enterica* cells without E20c was used as controls. Error bars are representative of ± SD of the three independent experiments performed in triplicates

Disruption of the integrity of the cell membrane results in efflux of small ions. Therefore, we evaluated the effect of different doses of E20c on the efflux of potassium ions from *S. enterica* cells. As shown in Fig. 6, treatment of *S. enterica* cells with E20c resulted in significant (p < 0.05) increase in extracellular concentration of potassium ions at all time points in a dose-dependent manner. Efflux of potassium ions was observed within 2 min of the addition of E20c that peaked to 16.3 and 8.4 parts per million (ppm) at concentrations 1.0 and 0.5 µg/ml, respectively at 6 min, after which the effect plateaued (Fig. 6).

### Checkerboard titrations

The antibiotic susceptibility profile of *S. enterica* MTCC 733 used in the study was determined by standard Kirby Bauer method that showed that it was resistant to number of antibiotics in different classes (Additional file 1, Table S1). Fast emerging antibiotic resistance among *S. enterica* strains is a serious problem that warrants novel alternatives. One of the alternatives could be to use bacteriocins as adjuncts to lower the therapeutic doses of antibiotics. Thus, interaction of E20c with conventional antibiotics and its effect on lowering the MIC of antibiotics for *S. enterica* was studied by using checkerboard titrations. Interestingly, as shown in Table 4, the MIC of E20c for *S. enterica* was lower than the MIC of all the tested antibiotics. Further, checkerboard titrations revealed that E20c had synergistic interaction with ciprofloxacin and the tested ß-lactams viz. ampicillin, penicillin and ceftriaxone as the fractional inhibitory concentration index (FICI) obtained in combination with E20c in all the cases was less than 0.5. E20c lowered the MIC of ciprofloxacin by 12.8 times and that of the three tested ß-lactams in the range 6.4–13.1 (Table 4). However, in combination with carbenicillin, E20c showed indifference.

**Table 4:** MIC and FICI of E20c alone and in combination with antibiotics against *S. enterica*

Antibiotic	MIC (µg/ml)			FIC of antibiotic	FIC of enterocin 20c	FICI antibiotic + E20c	Fold decrease in MIC of antibiotic
	Antibiotic	E20c	Antibiotic/E20c				
Ampicillin	12.5	0.5	1.81/0.07	0.14	0.14	0.28	6.9
Penicillin	25	0.5	1.9/0.156	0.08	0.32	0.39	13.1
Ceftriaxone	43.5	0.5	6.7/0.07	0.15	0.14	0.29	6.4
Ciprofloxacin	400	0.5	31.2/0.15	0.078	0.31	0.38	12.8
Carbenicillin	21.7	0.5	6.5/0.15	0.29	0.31	0.60	3.3

FICI = FIC of antibiotic + FIC of E20c = (MIC of antibiotic in combination/MIC of antibiotic alone) + (MIC of E20c in combination/MIC of E20c alone). The interaction was interpreted as follows: synergy, FICI ≤ 0.5; indifference, 0.5 < FICI < 2; and antagonism, FICI > 2. The values are mean ± SD of experiments performed in triplicate

## Discussion

In this study, enterocin E20c was purified from the CS of probiotic strain *E. hirae* 20c. *E. hirae* 20c was selected for the purification of E20c as its CS inhibited the growth of several Gram-negative bacterial pathogens, and the strain possessed good probiotic properties with no known virulence factors or genes [15]. The purified E20c was obtained as a single peak with the molecular weight of 6.5 kDa. The peak was further characterized by MALDI TOF/TOF MS and Pmf analysis. MASCOT search of the peptides obtained by trypsin digestion did not matched with any of the known bacteriocin; but it shared 34% homology with a hypothetical protein of *E. faecalis*. The sequence of the hypothetical protein was searched by Phobius software [16] to determine the presence of signal peptide that indicates the secretory nature of the protein. Results showed that the hypothetical protein possessed 41 amino acid long signal sequence (data not shown) that indicated its secretory nature. One of the peptide fragment of E20c matched with the signal peptide sequence of the hypothetical protein thereby indicating the secretory nature of E20c. The physico-chemical characterization of E20c was done that showed that it was both heat stable and stable at pH ranging between 2 and 8. The antimicrobial spectrum of E20c showed that it was active against both Gram-positive and Gram-negative pathogens but not against commensal lactobacilli isolated from the stool samples of healthy individuals. The antibiotic susceptibility profiles (Additional file 1) of *S. enterica*, *Sh. flexneri* and *Es. coli* strains used in the current study revealed that all the three pathogens could be MDR strains as they were resistant to at least one agent in more than 3 different antibiotic classes. Thus, enterocin 20c inhibited the growth of MDR Gram-negative human gut pathogens but not that of commensal gut lactobacilli spp.

Further, literature search revealed that only two enterocins, i.e., hiracin JM79 and LD3 have been earlier reported from *E. hirae.* Hiracin JM79 of molecular weight 8.157 kDa had antimicrobial activities against Gram-positive bacteria only, including various species of *Lactobacillus*, *Enterococcus*, and *Pediococcus* [17]. Whereas*, en*terocin LD3 (6 kDa) inhibited Gram-positive bacteria such as *Enterococcus*, *Lactococcus* spp. including lactobacilli, and Gram-negative bacteria, including *S. typhi* [18]. E20c, in contrast, had no activity against human commensal lactobacilli. Commensal gut lactobacilli are beneficial to human health owing to the number of important functional properties; therefore, their inhibition may lead to gut dysbiosis [19].

Further, time-kill studies were performed to determine the mode of action of E20c against *S. enterica*. Antimicrobial agents are categorized as bactericidal if they result in > 3 log decrease in CFU/ml [20]. Treatment of *S. enterica* cells with E20c at a dose of 1 µg/ml, resulted in a time-dependent decrease in the viable cell counts with 5 log10 CFU change within 60 min. This indicated the bactericidal mode of action of the enterocin. Similar results were obtained for bacteriocin BM1157 which caused more than 3 log10 decrease in CFU of *Lis. monocytogenes* after 30 min treatment [21]. In another study, pentocin JL-1 treatment resulted in 4.5 log10 decrease in CFUs of *Staph. aureus* cells within 60 min [22]. As bacteriocins are known to kill the target cell by permeabilizing their cell membrane due to their cationic nature, the effect of E20c on the cell membrane permeability of *S. enterica* cells was determined by flow cytometry and confocal microscopy. E20c treatment of *S. enterica* cells resulted in membrane destabilization that led to enhanced uptake of PI within the cells as shown by increase in their fluroscence with time. These results are similar to that reported for other bacteriocins such as nisin [23] and lactacin F [24]. Further, the SEM images of E20c-treated *S. enterica* clearly showed cell surface damaging effects such as cell shrinkage and surface indentations due to the loss of cell turgidity or membrane damage. Disruption of cell membrane integrity is associated with efflux of small molecules such as potassium ions and ATP, and large molecules such as proteins depending on the size of the pore [25]. Our results showed that E20c disrupted the cell membrane integrity of *S. enterica* cells resulting in efflux of potassium ions in a dose-dependent manner. Other enterocins such as enterocin P [26] and enterocin CRL35 [27] are also known to induce the efflux of potassium ions from the target bacterial cell.

Conventional antibiotics such as ampicillin and ciprofloxacin are commonly used for the treatment of *S. enterica* infections. However, resistance to these antibiotics limits the therapeutic options. The antibiotic susceptibility profile of *S. enterica* used in this study indicated that the strain was resistant to a number of antibiotics in the classes β-lactam, fluoroquinolone, tetracycline and aminoglycoside (Additional file 1; [28]). MIC of penicillin for *S. enterica* strain used in the current study was 25 µg/ml and that of ciprofloxacin was 400 µg/ml. Next, we determined the ability of E20c to lower the MIC of resistant antibiotics. Checkerboard titrations was revealed synergistic interaction between E20c and conventional antibiotics such as ampicillin, penicillin, ceftriaxone and ciprofloxacin. Treatment of *S. enterica* cells with conventional antibiotics in the presence of E20c lowered the MIC of antibiotics in the range 6.4 (ceftriaxone) to 13.1 (penicillin). However, no synergism was observed with carbenicillin. Few other studies have reported the synergistic effect of purified bacteriocins with antibiotics. For example, Singh et al. [29] showed the synergistic effect of nisin in combination with ampicillin, ceftriaxone, and cefotaxime against the clinical strains of *S. enterica* serovar Typhi. Enterocin CRL35 at a very low dose of 4 ng/ml showed synergy with tetracycline, erythromycin, and chloramphenicol but not with ciprofloxacin and ampicillin against *Lis*. *innocua* [30]. In another study, enterocins DD28 and DD93 at the dose of 50 µg/ml synergized with erythromycin and kanamycin against methicillin-resistant *Staph. aureus* [31].

The *S. enterica* strain used in the present study showed resistance to antibiotics penicillin, methicillin and ciprofloxacin. ß-lactam antibiotics are known to enter Gram-negative bacterial cells through porins; whereas, fluoroquinolones enter through both porins and lipid-mediated pathways [32]. Therefore, one of the mechanism of ß-lactam and ciprofloxacin resistance used by Gram-negative bacteria includes loss or severe reduction in the numbers of porins, or mutation leading to reduced permeability of porins [33]. Thus, the mechanism by which E20c synergized with these antibiotics may be explained by their ability to enhance the permeability of the outer membrane to the antibiotics thereby reversing their resistance and reducing their MIC. Another study that evaluated the combinations of nisin-cefotaxime and nisin-ceftriaxone against *S. enterica* showed that the combination of bacteriocin-β-lactams significantly increased (p < 0.05) the cell membrane permeability of the treated cells to hydrophobic fluorescent probe, 1-N-phenylnaphthylamine [29]. Further, they also observed dose- and time-dependent inhibition of DNA, RNA and protein synthesis in the presence of the tested combinations.

## Conclusion

E20c appears to be a novel low molecular weight enterocin having bactericidal activity against several Gram-negative bacteria due to its cell membrane-permeabilising action. It is a promising candidate for standalone and adjunct therapy against *S. enterica* infections. With fast emerging problem of multidrug resistance in *Salmonella*-related infections, enterocin E20c can serve as an effective therapeutic option that should be further evaluated in animal models.

## Methods

### Bacterial strains

*E. hirae* 20c used for the purification of enterocin was obtained from the vaginal microflora of healthy women as reported previously [15]. Commensal *Lactobacillus* spp. used as indicator strains were isolated from the stool samples of healthy children and the method for the isolation and characterization were reported in the previous study [34]. All the LAB strains were cultured in De-Man Rogosa and Sharpe (MRS) media at 37 °C in anaerobic jars. All the chemicals and bacterial growth media used in this study, except where mentioned, were purchased from HiMedia Laboratories Pvt. Ltd. (Mumbai, India).

All the indicator pathogenic strains used in the study were procured from MTCC, Institute of Microbial Technology, Chandigarh, India. The pathogenic indicator strains, except *M. smegmatis* were propagated in Brain heart infusion (BHI) broth at 37 °C under aerobic stationary conditions. *M. smegmatis* was cultured in the 7H9 broth supplemented with Middlebrook OADC Growth Supplement containing bovine serum albumin fraction V and Tween 80 under aerobic conditions at 37 °C.

### Agar gel diffusion assay

The antimicrobial activities of E20c against various indicator pathogenic bacterial strains was determined by modified agar gel diffusion assay [35].

### Purification and characterization of E20c

E20c was purified from the CS of *E. hirae* 20c by using ammonium sulphate precipitation followed by cation-exchange chromatography. Briefly, 1 litre of MRS broth was inoculated with 2% of overnight grown culture of *E. hirae* 20c and incubated at 37 °C for 16 h in anaerobic jars. To prepare the CS, culture was centrifuged at 10,000 rpm at 4 °C. The proteins in the CS were precipitated by adding ammonium sulphate till 60% saturation (w/v) at 4 °C. The protein precipitates were separated by centrifugation (10,000 rpm at 4 °C) and dissolved in sodium acetate buffer (20 mM; pH 4.5). The dissolved precipitates were further desalted by passing through Biogel PD-10 column (GE HealthCare, USA) equilibrated with sodium acetate buffer. The active fractions obtained from Biogel PD-10 column were pooled and applied on SP-Sepharose Fast Flow cation-exchange column (50 × 10 mm; GE Healthcare) and eluted with a linear salt gradient of 0.1 to 1 M NaCl in sodium acetate buffer. The fractions were tested for the antimicrobial activity against various indicator pathogens by agar gel diffusion assay. The active fractions were lyophilized and dissolved in MilliQ water. At every purification step, protein concentration was determined by using Bradford’s method [36].

Denaturing gel electrophoresis was carried out under reducing conditions to determine the molecular weight of the purified E20c [37]. Protein separation was carried out in 15% (w/v) polyacrylamide separating gel and 6% stacking gel. After electrophoresis, one lane of the gel was cut and stained with silver nitrate (SRL, India) whereas, the other lane of the gel was subjected to agar gel overlay assay against *S. enterica* in BHI soft agar [38].

### MALDI TOF/TOF MS

The silver-stained gel bands were cut, destained, trypsinized, extracted and subjected to MS by using MALDI-TOF/TOF-Proteomics Analyzer (UltrafleXtremeTM mass spectrometer; Bruker Daltonics Inc. Germany). A combined MS and LIFT-MS/MS were performed using BioTools 3.0 software (Bruker Daltonics Inc. Germany). The TOF spectra were recorded in positive ion reflector mode with a mass range from 700 to 3500 Da. Five hundred shots were accumulated for each spectra. Two most abundant peptide ions were then subjected to fragmentation analysis to determine the peptide sequence. Database search was performed using MASCOT search engine (Version 2.1) and NCBInr protein databases. The parameters used for search were as follows: taxonomy, Firmicutes; enzyme, trypsin; the fixed modification, carbamidomethyl (C); the variable modification, Glu- > pyro-Glu (N-term Q) and oxidation (M); parent ion mass tolerance at 50 ppm and MS/MS mass tolerance of 0.7 Da; one missed cleavage allowed. The identified proteins among the top hits on the search report with individual ions scores > 44 indicated identity or extensive homology (p < 0.05).

### Physico-chemical characterization of purified E20c

Thermostability of purified E20c, was determined by subjecting it at a concentration of 1 µg/ml to different temperatures for different time periods. For determining the pH sensitivity, the pH of E20c was set to different values between 2 and 10 and incubated at 37 °C for 1 h. The pH was reset at 6.5 and the antimicrobial activity was determined by agar well diffusion assay. Further, the effect of various proteolytic and lipolytic enzymes on E20c was determined. E20c was treated with enzymes proteinase K, trypsin, pepsin, and lipase (Sigma Aldrich, India) at the concentration of 1 mg/ml for 1 h at 37 °C, followed by heat inactivation at 60 °C for 10 min. The residual antimicrobial activity was determined by agar gel diffusion assay.

### Safety evaluation

Hemolytic activity of E20c was evaluated by hemoglobin release assay [39] against human RBCs. The defibrinated human blood was centrifuged at 1200 rpm for 15 min at 37 °C and RBC-containing pellet was suspended in 10 ml PBS (pH 7.2). RBC suspensions (500 µl) were incubated with 100 µl of different concentrations of E20c at 37 °C for 1 h. Thereafter, the suspensions were centrifuged at 3000 rpm for 5 min and the hemoglobin release in the supernatant was monitored by taking OD_415_. TritonX-100 (1%) treated and PBS-treated RBCs were used as positive and negative control, respectively. The percentage of RBC lysis was calculated by using the equation: (A_T_–A_C_)/(A_X_–A_C_) × 100.

Where A_T_ is OD_415_ of E20c-treated RBCs, A_C_ is OD_415_ of PBS treated-RBCs and A_X_ is OD_415_ of 1% tritonX-100-treated RBCs.

### Determination of MIC and time-kill studies

MIC of E20c and conventional antibiotics against *S. enterica* was determined by broth dilution method [40]. Time-kill assay was performed as per the protocol by Joshi et al. [41] with slight modifications. E20c at the concentration of 2× MIC was added to 1 ml of BHI and inoculated with 10^7^ CFU/ml of *S. enterica* cells. The cells were incubated at 37 °C and 100 µl of the culture at different time points were serially-diluted and plated on nutrient agar plates for viable cell counting.

### Effect of E20c on the cell membrane permeability

The effect of E20c on cell membrane integrity was studied by using a flow cytometric method [42] and confocal microscopy with slight modifications. *S. enterica* cells were suspended in PBS at a concentration of 1 × 10^6^ CFU/ml. E20c (1 µg/ml) was added to the cell suspension and incubated at 37 °C for 15 and 30 min. The cell suspension was centrifuged (10,000 rpm; 5 min) to obtain cell pellet that was treated with PI (1 µg/ml) and incubated for 15 min at 4 °C under dark. Fluorescence of *S. enterica* cells was monitored by running the cells through flow cytometer (Accuri C6 Flow Cytometer) in FL2 channel. Data was analyzed by using C Flow Plus software (Becton–Dickinson, San Jose, CA, USA). Simultaneously, 10 µl of PI-stained cell suspension was placed on the glass slides, fixed with 5 µl Flourmount solution (Sigma) and viewed under a confocal microscope (Nikon, A1R).

### Scanning electron microscopy studies

SEM was performed to view the morphological changes induced in the E20c-treated *S. enterica* cells. Overnight grown culture of *S. enterica* at OD_595_ of 0.3 was treated with E20c (1 µg/ml) for 60 min at ambient temperature. After 60 min, the cells were centrifuged at 10,000 rpm for 5 min and re-suspended in PBS. Untreated *S. enterica* cells were used as control. The cell suspensions were placed on the glass coverslips and dehydrated according to the method by Kalab et al. [43]. Silver sputtering was done and the stubs were examined under SEM-EVO LS-10 (Carl Zeiss, Germany).

### Efflux of potassium ions

Destabilization of the cell membrane results in efflux of small ions. Therefore, we evaluated the effect of different doses of E20c treatment of *S. enterica* on the extracellular potassium ion concentration [44]. The bacterial cells grown till mid-log phase were centrifuged (10,000 rpm; 5 min) to obtain cell pellet, washed twice and suspended in 2.5 mM sodium HEPES (4-(2-hydroxyethyl)-1-piperazineethanesulfonic acid) buffer (pH 7.0) to obtain an OD_595_ of 1.0. Purified E20c was added to the cell pellets of *S. enterica* in two separate tubes to obtain final concentrations of 1 µg/ml and 0.5 µg/ml. Samples (1 ml) were taken at intervals of 2, 4, 6, and 8 min and immediately chilled on ice. *S. enterica* cells without E20c was used as controls. The samples were filter sterlised (0.2 μ) to separate the cells and the potassium ion concentration in the supernatants was determined by flame photometry (Systronics 128, Gujarat, India). The experiment was performed thrice in triplicates.

### Checkerboard titrations

Interaction of E20c with conventional antibiotics was determined by using checkerboard titration method [45]. Each antimicrobial was used at tenfold higher concentration than its MIC and diluted to test concentrations higher, equal and lower than MIC. Two-fold serial dilutions of the combination of antibiotics with E20c were made in 100 µl Mueller–Hinton broth in 96-well microtitre plate. Overnight grown culture of *S. enterica* was diluted using sterile BHI broth to obtain OD_595_ of 0.1 (10^8^ log10 CFU/ml). Five microlitres (5 × 10^5^ log10 CFU) of the culture suspension was added to each well and the plate was incubated at 37 °C for 24 h and observed for visual turbidity. The FICI was calculated as follows: FICI = FIC of antibiotic + FIC of E20c, where FIC of antibiotic is the MIC of antibiotic in the combination/MIC of antibiotic alone, and FIC of E20c is the MIC of E20c in the combination/MIC of E20 alone. FICI ≤ 0.5 indicate synergy; 0.5 < ΣFIC < 2 indicate Indifference; and ΣFIC > 2 indicate antagonism.

## Supplementary information

**Additional file 1: Table S1:** Antibiotic susceptibility profile of *Salmonella enterica* MTCC 733**. Table S2:** Antibiotic susceptibility profile of *E. coli* MTCC119. **Table S3:** Antibiotic susceptibility profile of *Shigella flexneri* MTCC1457.

## Data Availability

All data generated or analysed during this study are included in this published article.

## Abbreviations

CS: Culture supernatant
LAB: Lactic acid bacteria
MRS: De-Man Rogosa and Sharpe
MIC: Minimum inhibitory concentration
FIC: Fractional inhibitory concentration
RBC: Red blood cells
WHO: World health organization
BHI: Brain heart infusion media
CFU: Colony forming units
PBS: Phosphate buffered saline
MS: Mass spectrometry
PMF: Peptide mass fingerprinting
HEPES: 4-(2-hydroxyethyl)-1-piperazineethanesulfonic acid
