# Supplementary material for: Novel enterocin E20c purified from Enterococcus hirae 20c synergised with ß-lactams and ciprofloxacin against Salmonella enterica
Source: Microb Cell Fact. 2020 May 4;19:98. doi: 10.1186/s12934-020-01352-x (PMC7197179; doi:10.1186/s12934-020-01352-x)
Supplement: Supplementary file 1 — Additional file 1: Table S1: Antibiotic susceptibility profile of Salmonella enterica MTCC 733. Table S2: Antibiotic susceptibility profile of E. coli MTCC119. Table S3: Antibiotic susceptibility profile of Shigella flexneri MTCC1457. [file 12934_2020_1352_MOESM1_ESM.docx]

**Additional file 1**

**Table S1:** Antibiotic susceptibility profile of *Salmonella enterica* MTCC 733

| **Antibiotic** | **Concentration (µg/ml)** | **Zone of inhibition(mm)** | **Susceptibility** |
| --- | --- | --- | --- |
| **ß-lactams** |  |  |  |
| Penicillin | 2U | 17±0.1 | R |
| Ampicillin | 10 | 33±0.09 | S |
| Methicillin | 5 | -* | R |
| **Fluoroquinolones** |  |  |  |
| Ofloxacin | 5 | 7±0.12 | R |
| Ciprofloxacin | 5 | 16±0.71 | I |
| Norfloxacin | 10 | 20±0.06 | S |
| Gatifloxacin | 5 | 18±0.11 | S |
| Sparfloxacin | 5 | - | R |
| Moxifloxacin | 5 | 17±0.32 | R |
| **Macrolides** |  |  |  |
| Azithromycin | 15 | - | R |
| Clarithromycin | 15 | - | R |
| **Aminoglycosides** |  |  |  |
| Kanamycin | 30 | 9±0.02 | R |
| Streptomycin | 10 | - | R |
| Gentamicin | 120 | 20±0.89 | R |
| Amikacin | 30 | 22±0.61 | S |
| **Cephalosporins** |  |  |  |
| Cefexime | 5 | - | R |
| **Oxazolidinones** |  |  |  |
| Linezolid | 15 | 46±0.52 | S |
| **Others** |  |  |  |
| Colistin | 10 | - | R |
| Tetracycline | 30 | 20±0.2 | S |

-* no zone of inhibition observed

Kirby Bauer disc diffusion method was performed, and zones of inhibition were measured in mm. Standard interpretation of antimicrobial susceptibility tests was done in accordance with EUCAST standards (2018). **S:** Susceptible; **I:** Intermediate; **R:** Resistant.

**Table S**2: Antibiotic susceptibility profile of *Es. coli* MTCC119

| **Antibiotic** | **Concentration (µg/ml)** | **Zone of inhibition(mm)** | **Susceptibility** |
| --- | --- | --- | --- |
| **ß-lactams** |  |  |  |
| Penicillin | 2U | -* | R |
| Ampicillin | 10 | - | R |
| Methicillin | 5 | ND | - |
| **Fluoroquinolones** |  |  |  |
| Ofloxacin | 5 | 14±0.37 | R |
| Ciprofloxacin | 5 | - | R |
| Norfloxacin | 10 | - | R |
| Gatifloxacin | 5 | 15±0.21 | S |
| Sparfloxacin | 5 | - | R |
| Moxifloxacin | 5 | 11±0.28 | R |
| **Macrolides** |  |  |  |
| Azithromycin | 15 | - | R |
| Clarithromycin | 15 | - | R |
| **Aminoglycosides** |  |  |  |
| Kanamycin | 30 | ND | R |
| Streptomycin | 10 | - | R |
| Gentamicin | 120 | 18±0.31 | S |
| Amikacin | 30 | 8±0.11 | R |
| **Cephalosporins** |  |  |  |
| Cefexime | 5 | - | R |
| **Oxazolidinones** |  |  |  |
| Linezolid | 15 | - | - |
| **Others** |  |  |  |
| Colistin | 10 | - | R |
| Tetracycline | 30 | - | R |

-* no zone of inhibition observed

Kirby Bauer disc diffusion method was performed, and zones of inhibition were measured in mm. Standard interpretation of antimicrobial susceptibility tests was done in accordance with EUCAST standards (2018). **S:** Susceptible; **I:** Intermediate; **R:** Resistant.

**Table S3:** Antibiotic susceptibility profile of *Shigella flexneri* MTCC1457

| **Antibiotic** | **Concentration (µg/ml)** | **Zone of inhibition(mm)** | **Susceptibility** |
| --- | --- | --- | --- |
| **ß-lactams** |  |  |  |
| Penicillin | 2U | -* | R |
| Ampicillin | 10 | - | R |
| Methicillin | 5 | ND | - |
| **Fluoroquinolones** |  |  |  |
| Ofloxacin | 5 | 15±0.12 | R |
| Ciprofloxacin | 5 | 15±0.53 | R |
| Norfloxacin | 10 | 14±0.10 | R |
| Gatifloxacin | 5 | 19±0.11 | R |
| Sparfloxacin | 5 | 13±0.45 | R |
| Moxifloxacin | 5 | 12±0.30 | R |
| **Macrolides** |  |  |  |
| Azithromycin | 15 | - | R |
| Clarithromycin | 15 | 9±0.22 | R |
| **Aminoglycosides** |  |  |  |
| Kanamycin | 30 | - | R |
| Streptomycin | 10 | - | R |
| Gentamicin | 120 | 10±0.19 | R |
| Amikacin | 30 | 13±0.10 | R |
| **Cephalosporins** |  |  |  |
| Cefexime | 5 | - | R |
| **Oxazolidinones** |  |  |  |
| Linezolid | 15 | - | R |
| **Others** |  |  |  |
| Colistin | 10 | - | R |
| Tetracycline | 30 | 13±0.42 | R |

-* no zone of inhibition observed

Kirby Bauer disc diffusion method was performed, and zones of inhibition were measured in mm. Standard interpretation of antimicrobial susceptibility tests was done in accordance with EUCAST standards (2018). **S:** Susceptible; **I:** Intermediate; **R:** Resistant.
